# Closed-Loop Transcranial Electrical Neurostimulation for Sustained Attention Enhancement: A Pilot Study towards Personalized Intervention Strategies

**DOI:** 10.3390/bioengineering11050467

**Published:** 2024-05-08

**Authors:** Emma Caravati, Federica Barbeni, Giovanni Chiarion, Matteo Raggi, Luca Mesin

**Affiliations:** Mathematical Biology and Physiology, Department Electronics and Telecommunications, Politecnico di Torino, 10129 Turin, Italy; emmacaravati12@gmail.com (E.C.); barbenifederica@gmail.com (F.B.); Giovanni.Chiarion@chuv.ch (G.C.); matteo.raggi@polito.it (M.R.)

**Keywords:** adaptive, transcranial direct current stimulation, closed loop, EEG, entropy, neurostimulation, sustained attention

## Abstract

Sustained attention is pivotal for tasks like studying and working for which focus and low distractions are necessary for peak productivity. This study explores the effectiveness of adaptive transcranial direct current stimulation (tDCS) in either the frontal or parietal region to enhance sustained attention. The research involved ten healthy university students performing the Continuous Performance Task-AX (AX-CPT) while receiving either frontal or parietal tDCS. The study comprised three phases. First, we acquired the electroencephalography (EEG) signal to identify the most suitable metrics related to attention states. Among different spectral and complexity metrics computed on 3 s epochs of EEG, the Fuzzy Entropy and Multiscale Sample Entropy Index of frontal channels were selected. Secondly, we assessed how tDCS at a fixed 1.0 mA current affects attentional performance. Finally, a real-time experiment involving continuous metric monitoring allowed personalized dynamic optimization of the current amplitude and stimulation site (frontal or parietal). The findings reveal statistically significant improvements in mean accuracy (94.04 vs. 90.82%) and reaction times (262.93 vs. 302.03 ms) with the adaptive tDCS compared to a non-stimulation condition. Average reaction times were statistically shorter during adaptive stimulation compared to a fixed current amplitude condition (262.93 vs. 283.56 ms), while mean accuracy stayed similar (94.04 vs. 93.36%, improvement not statistically significant). Despite the limited number of subjects, this work points out the promising potential of adaptive tDCS as a tailored treatment for enhancing sustained attention.

## 1. Introduction

Sustained attention refers to the ability of self-directed maintenance of cognitive focus for a continuous amount of time without being distracted, under non-arousing conditions [1]. The ability to maintain attention is a crucial aspect of daily functioning, both in the private sphere (e.g., engaging in conversations [2] and driving a vehicle [3]) and in professional settings (e.g., working on a computer [4] and studying [5]). Furthermore, as a fundamental cognitive function, sustained attention serves as the foundation for other cognitive domains, including executive functions, memory, and learning [6]. In addition, sustained attention performance is strictly linked to the activation of brain cortical networks, especially in the right hemispheric prefrontal and parietal regions [7,8]. Therefore, enhancing sustained attention could potentially lead to overall improvements in various aspects of cognition. For this purpose, determining the underlying brain networks mediating it is crucial in understanding the mechanisms that govern this cognitive process [6]. These aspects, with particular reference to the neural substrates and network dynamics underlying sustained attention, can be effectively investigated by exploring functional connectivity [9]. This examines how different brain regions communicate and coordinate their activities to support sustained attention [9], providing insights into various neuropsychiatric disorders characterized by alterations in attentional functions [1,10].

Sustained attention is typically evaluated and studied during tasks involving subjects responding to specific stimuli over extended periods (i.e., more than 10 min) [11]. Changes in sustained attention are measured both as fluctuations [12] and as decrement [13] in performance on these tasks over time. This decrement can be appreciated in different forms, such as a gradual decline in response accuracy, a higher percentage of errors, and a slowdown in reaction times. By quantifying the performance decrement, researchers can examine the endurance of sustained attention and identify factors that affect its maintenance or decline [6].

To enhance cognitive processes in both individuals with typical cognitive function and those with cognitive impairments, various methods have been explored. Recent studies highlighted the importance of the mental tasks used to elicit and modulate cerebral activity [14,15]. Other studies have investigated the application of non-invasive brain stimulation methods, with a particular focus on transcranial direct current stimulation (tDCS) [16,17], which enables the controlled experimental manipulation and reversible modulation of neuronal activity. It achieves this by delivering low-intensity currents (0.1–2.0 mA) through one or more active electrodes, generating an electric field that can interact with neural processing, altering plasticity and ultimately influencing relevant behaviors, based on the specific brain region involved, and the electrical current intensity and polarity. This current propagates through the soft tissues and the scalp, returning via the reference electrode (cathode) to close the circuit [18,19,20,21]. It is worth noting that the effect of the stimulation on neurons differs according to the type of polarity. Indeed, an anodal current increases cortical excitability, while a cathodal one produces hyperpolarization, decreasing cortical excitability. A positive anodal current is generally assumed to transiently enhance behaviors connected with the cortical region beneath the targeted electrode, while a negative cathodal current inhibits them [22]. Moreover, as documented in different studies [23,24,25], volume conduction affects the actual distribution of the current, and most of the electrical field is found about midway and not directly under the electrodes.

To date, it is believed that tDCS could either enhance cognitive performance or alleviate symptoms across a spectrum of neurological conditions (e.g., epilepsy [26], stroke [27]) and neuropsychiatric disorders (e.g., neuropathic pain [28], depression [29]), depending on the specific cortical area being targeted. The influence of tDCS extends beyond the stimulation period, depending on the current intensity and duration. Short sessions induce transient excitability changes [30], while longer ones can have long-lasting effects [31]. In general, five main parameters affect the outcomes: current polarity, current density, electrode size and position (affecting electric field orientation), stimulation duration, and the excitability of the stimulated area [32]. However, discrepancies exist in the literature due to varying experimental protocols, targeted brain regions, and individual responses. Meta-analyses demonstrate contrasting results [33,34]. Possibly, these disparities could be attributed to the limitations of conventional brain stimulation systems, which rely on static parameters and offer minimal adaptability to the specific subject and the changing brain activity. This approach could limit their effectiveness, as the same parameters may produce different results depending on the user and the current brain state.

In response to these limitations, closed-loop neurostimulation is emerging as an innovative solution. Closed-loop systems are designed to continuously monitor a patient’s brain activity and adjust stimulation parameters accordingly, thus potentially providing personalized therapy that is both efficient and automatic [35]. Compared to traditional open-loop methods, closed-loop neurostimulation may significantly enhance clinical outcomes, reduce side effects, and boost overall efficacy [36,37]. For example, the feasibility of a closed-loop system was demonstrated in a proof-of-concept study involving six healthy subjects, considering the power distribution of the electroencephalogram (EEG) spectrum [38]. Similarly, a device capable of automatically adapting the stimulation by analyzing only the beta range of the EEG signal is discussed in [39]. However, by relying solely on the power distribution across different bandwidths, the informative content of the EEG signal is not fully utilized. For this reason, this paper also includes complexity metrics to explore brain activity and control an adaptive closed-loop tDCS to enhance sustained attention in healthy subjects. To the best of our knowledge, this is the first work that aims to develop a closed-loop system for this purpose, while most of the published studies analyze motor memory [40] and sleep [41]. Specifically, the effectiveness of adaptive tDCS is investigated in enhancing the performances of the users during the Continuous Performance Task-AX (AX-CPT) [42]. The hypotheses of our study are that the close-loop stimulation can increase accuracy in the task and reduce the reaction time (by counteracting mental fatigue), with respect to conditions in which the subjects are either not stimulated or tDCS is applied open-loop (i.e., with a fixed stimulation current intensity and location).

Our work is intended as a pilot study (applied on healthy subjects), but further deepening the investigation could also open interesting perspectives in the treatment of cognitive pathologies, e.g., Attention-Deficit/Hyperactivity Disorder (ADHD) [43] and autism [44].

## 2. Materials And Methods

### 2.1. Participants

This study involved ten healthy right-handed undergraduate students (3 females, 7 males; age 23.9 ± 0.6 years). All participants had either normal or corrected-to-normal vision and no history of any neurological or psychological disorder. The study was conducted in accordance with the Declaration of Helsinki and was approved by the Ethical Committee of the University of Turin (approval number 0125508). Both a written consent form and a “Transcranial electrical stimulation (tES) safety questionnaire” [45] (see Appendix A) were filled out before the beginning of the experiment. Subjects were asked not to consume caffeine, alcohol, drugs, supplements for memory/concentration, or medications inducing drowsiness (e.g., antihistamines, anxiolytics, etc.) within the 12 h preceding the experiment.

To verify the effective homogeneity among the candidates, a Go/No-go task [46] was performed. The test was structured into two parts: first, participants were instructed to respond to the “Go” stimulus when withholding their response to the “No-go” stimulus, while the second part exploits the reversal condition. Each stimulus lasted 500 ms, with an inter-stimulus interval (ISI) of 1500 ms. A total of 320 stimuli were presented, with 160 stimuli in each condition. Comparable results, in terms of the percentage of correct responses (>97%), were used as a selection test to ensure homogeneity in the participants.

### 2.2. Attention Demanding Task: AX-CPT

The Continuous Performance Task (CPT) is a neuropsychological test designed to measure complex attentional functions, including sustained attention and response inhibition, to assess various clinical conditions such as attention deficit disorder and schizophrenia [47]. In this study, the AX-CPT variant [42] was used. During this test, randomly selected letters of the English alphabet are sequentially presented on a screen individually. Participants were instructed to respond as fast and accurately as they could when the letter X appeared immediately after the letter A while inhibiting the response for all the other cases.

The task parameters were configured as follows: a 70% probability for AX, 10% for non-A followed by X (e.g., BX), 10% for A followed by non-X (e.g., AY), and 10% for non-A followed by non-X (e.g., BY). A total of 1800 random stimuli were presented (i.e., 900 trials) with each letter shown for 500 ms. For this protocol we used the open source software PEBL (Psychology Experiment Building Language [48]) version 2.1, providing stimuli to the candidates in white color over a black background. Overall the task lasted 15 min.

The following attention indicators were estimated from the responses obtained from this task: total number of correct responses to each trial; accurate answer rate (AR); omission errors (i.e., number of errors resulting from the failure to respond to the target trial AX); commission errors (i.e., number of errors resulting from responding incorrectly to the non-target trials, e.g., AY, BX, BY); mean reaction time (RT), and RT variability. Moreover, we computed the Inverse Efficiency Score (IES), defined as the ratio between RT and AR [49].

### 2.3. EEG Acquisition and Processing

**Figure 1:**
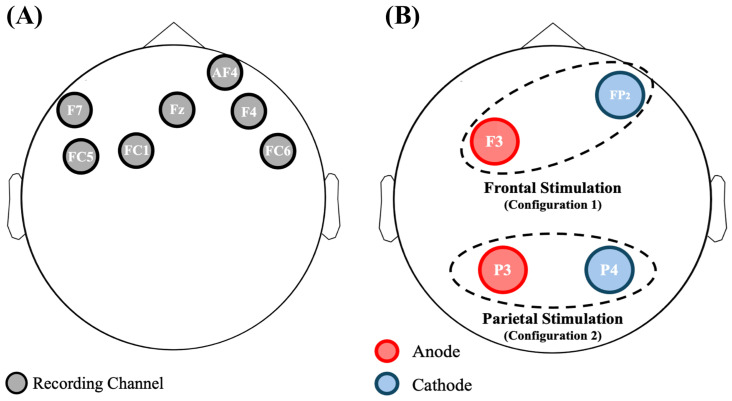
(**A**) Graphical representation of the electrodes used for recording the EEG signal. (**B**) Graphical representation of the two types of simulations used in this study: frontal (Configuration 1) and parietal (Configuration 2) stimulation, respectively.

For this study, we used the Starstim 8 (Neuroelectrics^®^, Barcelona, Spain), a wearable and wireless 8-channel system for both EEG acquisition and tDCS stimulation. Note that the channels used for the EEG recording could not be chosen for the tDCS stimulation. The EEG signals were sampled at 500 Hz with a resolution of 24 bit. During the whole experiment participants were seated and electrodes were secured with a neoprene cup based on the 10-10 standard. A reference electrode was situated near the right mastoid. Since one channel of the Starstim 8 was particularly noisy, the remaining ones were investigated: F7, FC5, FC1, Fz, AF4, F4, and FC6 (see Figure 1A).

**Figure 2:**
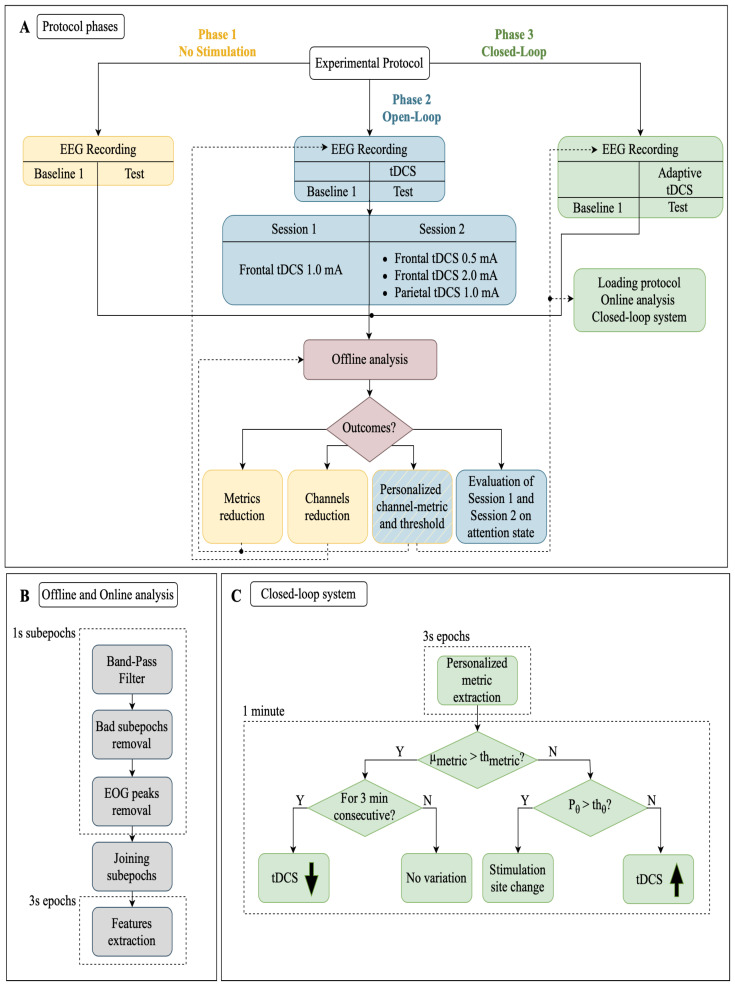
Block diagram of (**A**) protocol phases (Phase 1, Phase 2, and Phase 3); (**B**) offline and online data analysis procedures; (**C**) closed-loop system used in Phase 3 for personalized stimulation adaptation. $\mu_{metric}$: average value of the interested EEG metric measured during the Baseline condition—$th_{metric}$: personalized threshold estimated at the end of Phase 2—$P_{\theta }$: power of the EEG signal in the θ range (4–8) Hz—$th_{\theta }$: power in the θ range observed during the Baseline condition.

EEG signals were segmented into 1-second sub-epochs and filtered with a two-stage Chebyshev IIR filter, including a low-pass type 1 (cutoff at 30 Hz with 20 dB attenuation at 35 Hz) to remove power-line and high-frequency noises and a high-pass type 2 (cutoff at 4 Hz with 20 dB attenuation at 2 Hz) to mitigate slow drifts. The EEG pre-processing is graphically reported in Figure 2B. It is worth noting that this strategy benefits from low computational cost and reduced complexity, making it also suitable for the closed-loop strategy, where very short latency is required.

Then, noisy sub-epochs (containing noise spikes, system glitches, or electromyography (EMG) bursts) with amplitude exceeding a threshold of ±85 μV were identified and removed. To detect and remove eye-blink corrupted epochs, a fast and automated eye-blink detection algorithm based on Median Absolute Deviation (MAD) was implemented in MATLAB^®^. In particular, a noisy epoch was discarded if it contained at least one sample exceeding the threshold of 6 times its MAD (chosen by fine-tuning of few experimental data). Channel AF4 was specifically chosen as the reference for blink detection due to its frontal position, making it more susceptible to ocular artifacts.

To minimize EMG artifacts, a preventive approach was employed by instructing subjects to minimize movements, head shifts, talking, or position changes during the tests.

EEG signals were then segmented into non-overlapping 3-second epochs, from which different metrics were extracted, as detailed below.

### 2.4. EEG Metrics

Previous related studies demonstrated correlations between human attention and spectral features in the theta (θ, 4–8 Hz), alpha (α, 8–13 Hz), and beta (β, 13–30 Hz) frequency bands [50]. Consequently, these features were calculated starting from the 3-second epochs obtained from the power spectral density (PSD), estimated with Welch’s method (Hamming windows with 1 s duration and 50% of overlap) [51]. Therefore, we considered the powers in the frequency ranges between θ and β ($P_{\theta }$, $P_{\alpha }$, and $P_{\beta }$) also including the features mostly used for attention recognition, such as the power ratio features $P_{\beta /\theta }$, $P_{\beta /\alpha }$, and $P_{\beta /(\alpha +\theta )}$ (i.e., $P_{\beta }/P_{\theta }$, $P_{\beta }/P_{\alpha }$, and $P_{\beta }/\left(P_{\alpha }+P_{\theta }\right)$, respectively). In addition, the mean ($f_{mean}$) and median ($f_{median}$) frequencies were investigated as well.

Furthermore, given the inherent nonlinear properties of EEG data, analysis of complexity offers substantial potential in EEG research [52]. These methods allow researchers to investigate self-organization and pattern formation in the brain’s complex neural networks, offering deeper insights than traditional linear approaches. As a consequence, entropy metrics were included as well.

We investigated both single [53] and multiple time scales [54], encompassing the computation of Approximate Entropy (ApEn) [55], Sample Entropy (SaEn) [56], and Fuzzy Entropy (FuEn) [57] for single-scale assessments, as well as the computation of Multiscale Sample Entropy Index (MSEI) and Multiscale Fuzzy Entropy Index (MFEI) for multiscale evaluations [58,59]. As parameters, tolerance r=0.15 and embedding dimensions d=2 were chosen [60].

The best metrics for discriminating between concentration and relaxation states were selected through Fisher’s Ratio (FR), which takes into account the means of the distributions considered ($\mu_{1}$ and $\mu_{2}$) and their variances (${\sigma_{1}}^{2}$ and ${\sigma_{2}}^{2}$):

(1) $$FR=\frac{{\left(\mu_{1}-\mu_{2}\right)}^{2}}{{\sigma_{1}}^{2}+{\sigma_{2}}^{2}}$$

### 2.5. Statistical Analysis

The following statistical tests were performed to check the significance of the variations in AX-CPT results when using different stimulation paradigms. The following performance indexes were considered: accuracy, RT, RT variability, and IES. AX-CPT results were explored with a Wilcoxon Sign Rank test; *p*-values below 0.01 and 0.001 were considered significant and strongly significant, respectively.

### 2.6. Experimental Protocol

The primary objective was the development of a closed-loop tDCS-EEG system to enhance sustained attention during the cognitive AX-CPT test in healthy subjects. The experimental protocol was divided into three phases plus a final validation, each separated by a minimum period of one week and a maximum of one month. Considering the variability in research protocols, individual responses and potential residual stimulation effects, the selected interval aligns with the range reported in existing studies [32].

The first part (Phase 1) was focused on EEG recordings and served to detect the best couple of EEG metric and channel that manifested the most significant differences during sustained attention tasks, while the remaining ones (Phase 2 and Phase 3) exploited the tDCS-EEG combination during the tasks with an open-loop and closed-loop stimulation, respectively. A graphical representation of the experimental protocol is shown in Figure 2A. It is worth noting that, before the AX-CPT task, candidates observed 5 min of rest in a static posture with their eyes open. For the whole duration of this interval, referred to as “Baseline 1”, the EEG signals were recorded as well. The EEGs during Baseline 1 were used as a reference for the subsequent tasks. Each phase lasted approximately 20 min: 5 min for the Baseline 1 condition and 15 min to complete the AX-CPT test.

As reported in Figure 2A, Phase 2 was performed twice. That is because we investigated first the frontal tDCS with a stimulation current of 1.0 mA (Session 1) and then different current intensities in the frontal (0.5 and 2.0 mA) and parietal (1.0 mA) regions (Session 2).

In the next subsections, a more detailed description of the three phases is provided. It is worth noting that the three phases were completed in order.

### 2.7. tDCS Setup and Stimulation

As introduced in Section 2.3, the Starstim 8 device was used for this study. Taking into account the information available in literature [18,34], two possible configurations (graphically shown in Figure 1B) were chosen to personalize the stimulation.

- Frontal stimulation (Configuration 1): Active electrode in anodic stimulation at F3 and return electrode at Fp2.
- Parietal stimulation (Configuration 2): Active electrode for anodic stimulation at P3, and return electrode at P4.

Given the fixed size of the neoprene cup, it has been possible to guarantee replicability among the different phases of the protocol (described below) for the same subject (at least as a first approximation, as small displacements when repeating a test are unavoidable). Regarding the stimulation time, we stimulated the subjects for the whole duration of the AX-CPT tests (15 min) with either a fixed or adapted stimulation (open- and closed-loop, respectively).

#### 2.7.1. Phase 1

This phase aims to identify the channels that are mostly associated with a sustained attention state. This was accomplished through the implementation of a MATLAB^®^ algorithm that estimated metrics associated with the attentional states of healthy subjects. The implemented algorithm is based on prior research findings [61]. Note that in this phase all the available electrodes were exploited for EEG recording (as shown in Figure 1A).

EEG was processed and metrics were estimated, as detailed in Figure 2B and Section 2.3. Overall, a total of 13 metrics were investigated. The whole set of features was normalized by subtracting and then dividing by the median of the Baseline 1 values.

The metrics showing better discrimination between concentration and relaxation states in terms of FR were selected.

#### 2.7.2. Phase 2

The goal of Phase 2 is the identification of a personalized modality of stimulation. As reported in Figure 2A, this phase is divided into two sessions. Both are characterized by a fixed current tDCS during the AX-CPT test.

In the first session, a frontal stimulation (Configuration 1) with a current of 1.0 mA was applied during the cognitive task. By doing so, we evaluated whether an enhancement of attentional states due to stimulation occurs by comparing the metrics described in Section 2.4 during the AX-CPT test and Baseline 1 condition. Subjects were then divided into two groups (Group 1 and Group 2), based on changes in EEG metrics. This division enabled a rough distinction between those who responded well or not to the frontal stimulation, respectively. In particular, subjects in Group 2 showed an anomalous increase in the power of the theta rhythm during frontal stimulation, possibly reflecting mental fatigue [13] and/or immersion [62].

The purpose of the second session was to evaluate three possible changes in the stimulation strategy based on the assigned group. For Group 1 subjects, we stimulated in Configuration 1, exploring two current intensities: 0.5 and 2.0 mA. For Group 2, we investigated two different stimulation conditions: a current of 0.5 mA in the frontal region (Configuration 1) and a stimulation at 1.0 mA in the parietal region (Configuration 2). For both Phase 2 and Phase 3, the following channels of the Starstim 8 were used to record the EEG: AF4, FC6, and Fz.

Since the aim of the study is to adapt the stimulation to each individual subject, we derived the channel–metric pair that yielded the most favorable outcomes in each specific case. A classifier was developed to discriminate baseline and test values for each metric, using a threshold. Values exceeding the threshold were classified as test (1—positive class) and those below as baseline (0—negative class). For each experimental session, we selected the threshold that maximized the Youden’s index

(2) J=TPR+TNR−1

where TPR is the True Positive Rate and TNR is the True Negative Rate. The resulting accuracy values for each channel–metric pair were calculated using a confusion matrix.

This process was repeated across experimental sessions, considering only the ones that showed an increasing trend of the metrics during the test step compared to the baseline one. Then, accuracy in the AX-CPT was measured in this condition.

#### 2.7.3. Phase 3

The purpose of this phase was to evaluate the effects of closed-loop EEG-tDCS on sustained attention state, by modulating stimulation parameters based on the subject’s neural response.

Once the channel–metric pair was selected for each subject, the system initiated frontal stimulation at 1.0 mA and adapted the current intensity and stimulation site for each individual based on a personalized threshold system obtained in Phase 2 (see Figure 2C). The algorithm calculated the chosen metric every 3 s and assessed its deviation from the baseline after 20 epochs, allowing for minute-by-minute adjustments of stimulation parameters. This choice was made to balance neural response time with real-time system feasibility, considering the potential discomfort or disruption to the subject. If the mean of the estimated metric exceeded the threshold, we assessed whether this continued for three minutes. If so, then a current decrement occurred. When the estimated metric was below the personalized threshold, we compared the theta power of the signal (1-min length) with the theta threshold, fixed at 0.1 in this study. If the theta power of the signal exceeded the threshold, then the stimulation site was changed, otherwise a current decrement was applied.

#### 2.7.4. Validation

To assess the possible effects of familiarity with the cognitive task, a final acquisition (referred to as “Validation”) was recorded after the previous three phases. During this final step, we asked subjects to again perform the AX-CPT with the same conditions as Phase 1 (EEG acquisition only). Those last acquisitions, with a minimum delay of one week from Phase 3, were used to assess whether the volunteers improved their ability to perform the test (learning effect).

## 3. Results

**Figure 3:**
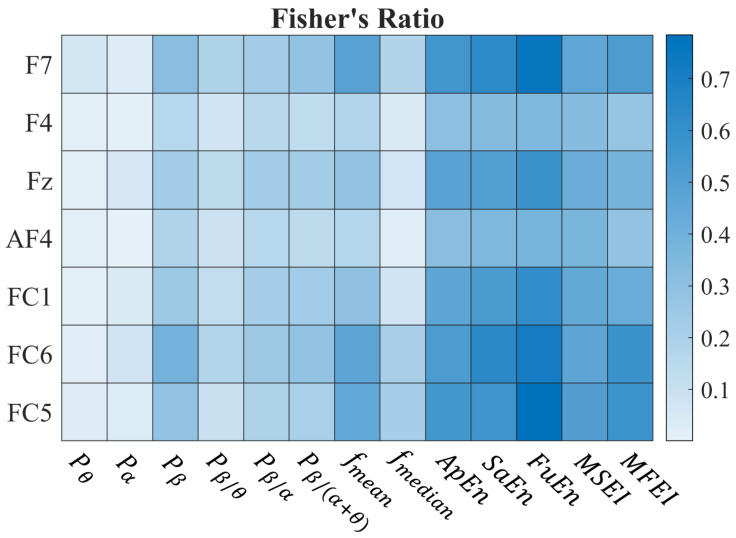
Results of Phase 1. The heatmap represents the median of the Fisher’s Ratios for each channel–metric pair across all subjects. The channels and the metrics are reported vertically and horizontally, respectively. A detailed explanation of the metrics explored can be found in Section 2.4.

We used Fisher’s Ratio (FR) to identify metrics that exhibit the greatest differentiation between the concentration and rest states (Phase 1). The results are reported in Figure 3 where a heatmap shows the median Fisher’s Ratio for each channel–metric pair across the whole set of subjects. The results show that entropy-based metrics exhibit high FR values for distinguishing relaxed and attentive states across all channels.

Therefore, we considered the FuEn and the MSEI, belonging to the single- and multi-scale classes, respectively. Indeed, according to Figure 3, they guarantee the highest FR in the respective classes. As two pairs of electrodes were kept available for either frontal or parietal stimulation and AF4 was used for blink detection, only two channels could be used for the EEG acquisition during the following phases (see Section 2.7.2): we chose FC6 and Fz.

**Figure 4:**
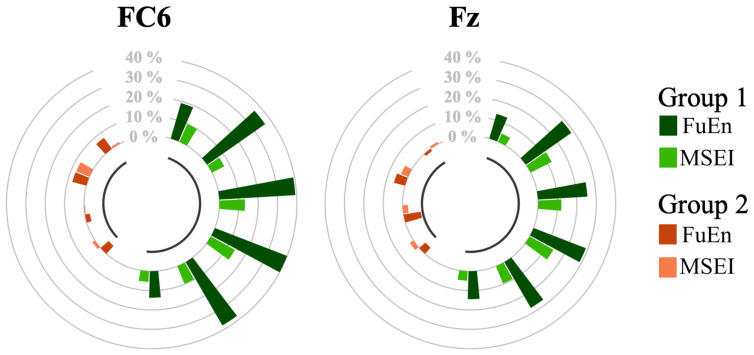
Results of Session 1 of Phase 2. Circle plots depict the impact of 1.0 mA frontal stimulation on channels FC6 and Fz. Each couple of bars is associated with a subject. Individuals belonging to Group 1 are colored light and dark green, while the ones belonging to Group 2 are identified with light and dark orange. Each bar represents the percentage increase (or decrease) in the metrics during the test with respect to the median value estimated in Baseline 1. Recall that FuEn and MSEI stand for Fuzzy Entropy and Multiscale Sample Entropy, respectively.

Note that we subtracted from each metric (estimated on 3 s of signal) its median value obtained during Baseline 1 ($m_{d}$) and we divided the resulting values by $m_{d}$. Restricting the analysis to the FC6 and Fz channels only, we performed Session 1 of Phase 2 (stimulation with 1.0 mA in the frontal region) on all the subjects estimating the FuEn and MSEI during the EEG acquisition. Therefore, four different channel–metric pairs were considered: FuEn–FC6, FuEn–Fz, MSEI–FC6, and MSEI–Fz. The results are reported in Figure 4, where we compare the median values of the metrics. Channels are considered separately and each pair of bars is associated with a subject.

The behavior of the subjects appears to be clustered into two groups. Specifically, not all the candidates enhanced the metrics due to the frontal stimulation at 1.0 mA (Configuration 1). Indeed, four subjects, classified in Group 2 (see Section 2.7.2), showed a variation of less than 10% on both channels. Among the subjects belonging to Group 1 (i.e., those who benefited from the frontal stimulation), channel FC6 was the most effective in discriminating attentional states, with FuEn showing the most substantial increase.

To examine the relationship between stimulation current intensity and the selected metrics, we explored different stimulation conditions as described in Section 2.7.2. To do so, half of the subjects received the treatment with a current of 0.5 mA and the remaining ones with a current of 2.0 mA.

An in-depth analysis was also conducted within Group 2 to explore the factors contributing to the absence of attentional state enhancement during stimulation. A closer examination of the metrics, including previously excluded spectral features, revealed an atypical increase in theta band power during the test step with tDCS in all four subjects within this group. As reported in Section 2.7.2, we modified both the current intensity and the region of stimulation, exploring different modalities to try to help Group 2 subjects improve their attentional state.

**Figure 5:**
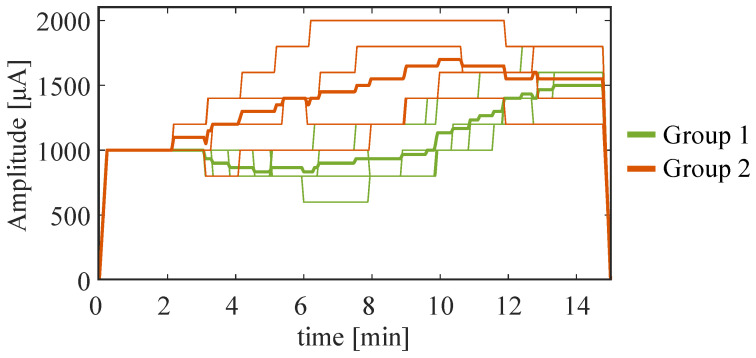
Results of the closed-loop simulation in Phase 3. Dynamic change in current intensity during the test phase for both Group 1 and Group 2. The thicker lines represent the average trend in each group, while the thinner ones are related to each subject.

In Phase 3, stimulation parameters were adapted to each participant based on their previously selected channel–metric pairs and responses, utilizing the closed-loop system described in Figure 2C. The temporal profiles of the stimulation current are presented in Figure 5, where the thicker lines represent the average trend in each group. Table 1 provides a comprehensive summary of the adapted stimulation protocols, detailing group, channel–metric pair, stimulation type, and maximum current intensity for each subject during the test.

In addition to the analysis of the results derived from the metrics extracted from the EEG signal acquisitions, a comprehensive assessment was performed on the outcomes of the AX-CPT test administered to the subjects.

**Figure 6:**
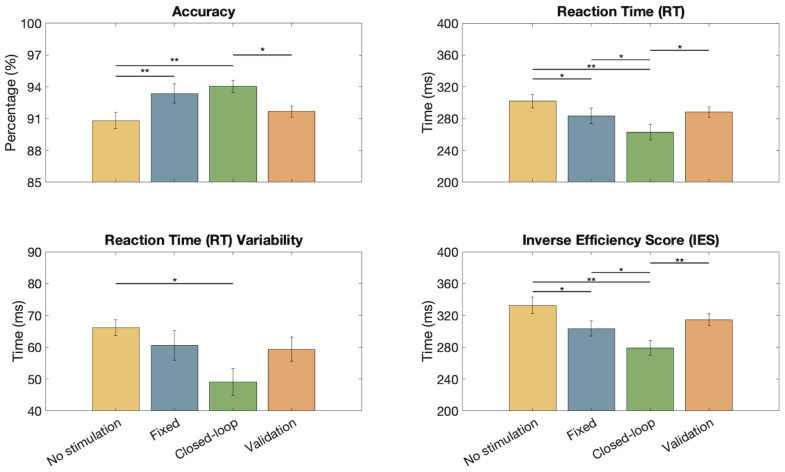
Average accuracy, reaction time (RT), RT variability, and Inverse Efficiency Score (IES) for the different conditions: initial test with no stimulation (yellow), fixed stimulation at 1 mA (blue), closed-loop (green), and final validation (orange). On each bar, the mean and standard error are shown. The significant results of the Wilcoxon Signed Rank test are reported for each pair (p<0.05 *, p<0.01 **).

In Figure 6, it is possible to appreciate the performances of the AX-CPT test during different stimulation conditions. The average values of accuracy, RT, variability in RT, and IES are shown with their respective standard errors. The results show a significant improvement in the accuracy (Wilcoxon Signed Rank test) when using an adaptive closed-loop stimulation instead of the no-stimulation conditions (p<0.01 and p<0.05 for the initial and the validation test, respectively). No significant differences were detected when comparing the accuracy of the adaptive and fixed stimulation. However, the adaptive closed-loop stimulation showed statistically significant reductions in the average RTs when compared to all the other conditions (p<0.05 in all cases except p<0.01 in the comparison with the first no-stimulation condition). Regarding RT variability, the closed-loop stimulation showed a significant improvement only in the comparison with the no-stimulation condition (p<0.05), still showing the smallest average value among the testing conditions. IES shows an improvement in performances during closed-loop stimulation with respect to all other conditions (with highly significant variation with respect to both conditions with no stimulation).

**Figure 7:**
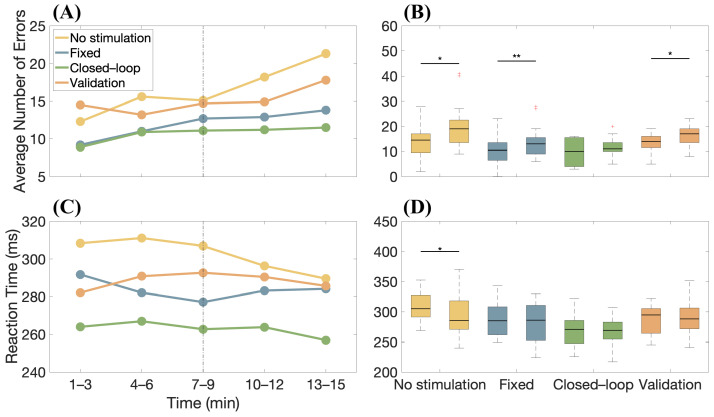
(**A**) Average error over time across subjects during the AX-CPT test, considering different phases of the experiment and stimulation conditions. The dashed line divides data into two parts relative to the first and last six minutes of the task. (**B**) Boxplots relative to the number of errors made by subjects in the different stimulation conditions during the first and the last six minutes of the experiment. The significant differences in the performances in the initial and final parts of the experiment are tested with the Wilcoxon Signed Rank method (p<0.05 *, p<0.01 **). (**C**) Same as (**A**), but considering the reaction time (RT). (**D**) Same as (**B**), but considering the RT.

Moreover, the overall trend of errors was assessed over time and the temporal variations in concentration stability across the different study phases are highlighted in Figure 7A, where the average number of errors made by the 10 subjects was calculated at 3-min intervals for each phase. Overall, an increase in errors in the final part of the protocol is observed. Figure 7B shows box-and-whiskers plots relative to the number of errors made by the candidates in different stimulation conditions considering the first and the last six minutes of the task. A Wilcoxon Signed Rank test showed a statistical decrease in performances, documenting the effect of fatigue on concentration during the task, with the exception of the case in which closed-loop stimulation was employed. With the same format as in Figure 7A,B, the variations in the average RT are shown over time in Figure 7C,D, respectively. Despite mental fatigue, RT is approximately constant (with the exception of the first test, for which an increase in the speed of reaction of the subjects is noticeable, as if they were becoming more familiar with it as time passed). We also notice that the participants were always faster (on average) when the adaptive stimulation was used.

## 4. Discussion

Neurostimulation can be useful in enhancing the attentive state. In this work, we explored whether a subject-specific and real-time adapted closed-loop stimulation can bring advantages over either a no-stimulation or a fixed-stimulation paradigm. We started by acquiring EEGs through the Starstim 8, a low-density system that can also be used for providing the tDCS stimulation sacrificing some electrodes for the EEG recordings. The first phase of the protocol aimed to find the best channel–metric pair associated with sustained attention during the AX-CPT task. Overall, the complexity metrics FuEn and MSEI resulted as the best among those investigated. This outcome is in line with [61], indicating that the complexity metrics are the most suited for determining the attention state in protocols involving EEGs.

The EEG channels less affected by artifacts and showing best discrimination between relaxed and concentration conditions were used to monitor the subjects during the tests and to control the adapted stimulation. The selected channels were FC6 and Fz, which is in accordance with [63] indicating that the frontal region plays an important role during attention tasks. AF4 was the reference channel for blink detection, due to its frontal position and susceptibility to ocular artifacts; considering its proximity to AF4 and low discrimination value, F4 was excluded. Moreover, left-side electrodes (FC1, FC5, and F7) were susceptible to EMG artifacts, especially given the participants’ right-handedness primarily engaging the contralateral hemisphere in motor activity; consider also that most of the informative content in EEG could be appreciated in the right hemisphere when dealing with sustained attention [7,8]. Additionally, these electrodes are positioned close to F3, which is used as an active electrode in subsequent phases, making them prone to stimulation artifacts.

Subjects were split into two groups, depending on their response to frontal stimulation observed during the first step of Phase 2. Indeed, as observed in Figure 4, some subjects showed on FC6 and Fz channels an increase in the complexity metrics chosen (FunEn and MSEI) after the 1.0 mA frontal stimulation (Group 1), whereas others did not (Group 2). This result is in line with the outcomes of previous studies [64,65], where other brain regions were shown to be involved in attention modulation in addition to the well-known frontal region [63]: one of them is the parietal region. Our outcomes partially confirm the results of [66] which indicate that the frontal region is a crucial neural hub for attention-related neurofeedback. However, further studies are necessary to verify this statement since both studies enrolled a restricted number of volunteers.

Group 2 subjects showed an increase in $P_{\theta }$ during frontal stimulation, suggesting that they manifested a specific state known as immersion, which is characterized by increased involvement [62]. Parietal stimulation was used as an alternative to frontal tDCS when the power of theta rhythm increased. In this way, the position of the stimulation constituted a further degree of freedom (in addition to the intensity of the stimulation current, and the EEG channel and metric) to better suit the subjects, particularly those we classified as Group 2. By analyzing the results of the tests during closed-loop stimulation (Phase 3), while Group 1 always received frontal stimulation, subjects in Group 2 consistently received parietal stimulation (Table 1). Concerning the intensity of stimulation, we also observed some difference across groups, with candidates of Group 1 showing a smaller tDCS than those of Group 2, but still converging to about 1.5 mA at the end of the test (Figure 5).

However, the most striking results concern the final performances (Figure 6). By analyzing data from the AX-CPT test, it is noticeable that the majority of errors tend to occur toward the end of the test (see Figure 7A). This pattern suggests a potential decline in concentration or the onset of mental fatigue as subjects progress through the task.

Specifically, adaptive neurostimulation can help the subject sustain mental activity. Indeed, there is a tendency for the number of errors to increase over time, on average (see Figure 7A), showing a decrease in attention due to fatigue. The smallest reduction in average performance is found in the case of closed-loop stimulation. Furthermore, comparing the number of errors made by the candidates at the beginning of the experiment (i.e., the first 6 min) and at the end (i.e., the last 6 min), performance decreases statistically for all conditions except in the case of closed-loop stimulation (see Figure 7B). This suggests that our subject-adapted stimulation protocol provides an aid in sustaining the demanding attentional task, being particularly effective in counteracting fatigue. The reaction time was also smaller (averaging across subjects) when using the closed-loop stimulation, compared to the other conditions (no stimulation or open-loop) and during the entire experiment (see Figure 7C,D).

Moreover, taking the overall performances, the closed-loop stimulation allowed increasing the accuracy (94.04%), and reducing the reaction time (262.93 ms) and its variability (49.04 ms), resulting in a smaller Inverse Efficiency Score (279.24 ms; notice that, despite the small dataset, the IES is also statistically smaller from the condition with fixed stimulation).

It is worth noting that in this study we did not investigate the causation between the channel–metric pairs in Phase 2 with the accuracy in the AX-CPT. However, the adaptation to the subject in closed loop allowed significant improvements in the performances (see Figure 6 and Figure 7). Thus, the hypotheses of our study stated at the end of the Introduction (i.e., improvements in accuracy and reaction time) have been partially confirmed: when using the proposed closed-loop stimulation, reaction times and IES decrease statistically and accuracy improves, but only on average when compared to open-loop stimulation. It is noteworthy that volunteers participating in this study were healthy university students. Therefore, future studies enrolling subjects with deficit disorders would be useful to define which channels to consider for applying our method. Indeed, it has been demonstrated that people suffering from attention disorders manifest hypoactivation in the frontal region [66], so it is possible to assume that different channels would be chosen from the ones we adopted for modulating the stimulation.

To the best of our knowledge, this is the first study focusing on the development of a closed-loop system for attention enhancement. Therefore, it is not possible to comprehensively compare our results with the literature. Overall, this study aims to demonstrate a possible application of this stimulation strategy as an effective tool to enhance the attention state. Our solution outperformed on average both the fixed- and no-stimulation conditions underscoring the potential benefits of adapted stimulation in providing a more stable and consistent performance over time.

### Limitations and Future Directions

This study shows an interesting potential application of tDCS in enhancing the performance of attention tasks. However, this work has different limitations. The study was conducted on a restricted sample and with a low-density system for EEG recordings only. When comparing the different stimulation modalities, the results do not look impressive. The stimulation improved the quality of the task by about 3% and reduced the reaction time by about 40 ms. The results of adaptive stimulation compared to a fixed current amplitude condition look even less significant. However, consider that we did not work on patients showing some cognitive impairments but on high-level university students in their final years of study, selected on the basis of good performances on a “Go/No-go” task. Their performances on the AX-CPT were very high (accuracy over 90% on average) and their reaction times were good even without any support from the stimulation. Thus, we could reasonably expect only marginal improvements. Future work is suggested including patients with cognitive deficits, where more room for improvement is expected.

Other future studies are suggested, involving a larger dataset (of either healthy subjects or patients) and high-density systems to investigate further the effects of our approach, test our hypotheses and analyze the brain activity with a higher resolution.

Subjects could be screened for cognitive status comprehensively before and after the tests.

Furthermore, it could be important to investigate the electric field distribution patterns in the brain and to study more deeply the effect of adaptive stimulation in different brain regions. Both the complex geometry and conductivity of the tissues (including the skull, the cerebrospinal fluid, etc.) affect the distribution of current induced by the stimulation and its effect on the brain.

## 5. Conclusions

This study describes an automatic closed-loop system for adaptive transcranial direct current stimulation (tDCS) to enhance sustained attention in an AX-CPT test. The main innovation is in the specific application associated with the adopted methodology for the closed-loop neurostimulation, involving complexity measurements as control parameters, and the possibility of changing both location and amplitude of the stimulation in real time. Despite a limited number of subjects, we obtained improvements over no-stimulation and fixed-tDCS conditions, both in terms of accuracy and reaction time. Moreover, the performances decrease over time, showing the effect of mental fatigue, when the subjects were either not stimulated or supported by a fixed tDCS. Closed-loop stimulation counteracted this effect by supporting the subjects in maintaining the focus on the task.

These preliminary results lay the basis for future analyses to explore the benefits of neurostimulation with tDCS. Specifically, to further improve the effectiveness of the closed-loop systems, future studies may consider the following directions: multi-sensor approaches (i.e., also considering heart rate or its variability) to find the optimal stimulation suited to the subject, the investigation of a large dataset (which could also be stratified with respect to participants’ demographics), a solid comparison with other non-invasive brain stimulation, and the effect of long-term stimulation. Once this validation on healthy subjects is obtained, the protocol could be extended to patients (e.g., with attention deficits) or integrated into cognitive enhancement programs.

## Figures and Tables

**Table 1:** Adapted stimulation: group, channel–metric pair, stimulation type, and maximum current intensity for each subject during the test.

	Subject	Channel–Metric Pair	Stimulation Site	Maximum Amplitude [μA]
Group 1	1	FC6–FuEn	Frontal	1800
	2	FC6–FuEn	Frontal	1400
	3	FC6–FuEn	Frontal	1600
	4	FC6–FuEn	Frontal	1600
	5	FC6–FuEn	Frontal	1600
	6	FC6–FuEn	Frontal	1200
Group 2	7	FC6–MSEI	Parietal	1800
	8	FC6–MSEI	Parietal	1400
	9	Fz–MSEI	Parietal	2000
	10	FC6–FuEn	Frontal/Parietal	1600

## Data Availability

Data are available from the corresponding author upon reasonable request.

## Abbreviations

The following abbreviations are used in this manuscript:

ADHD	Attention-Deficit/Hyperactivity Disorder
AR	Accurate Answer Rate
ApEn	Approximate Entropy
AX-CPT	Continuous Performance Task—AX
CPT	Continuous Performance Task
EEG	Electroencephalography
EMG	Electromyography
FR	Fisher’s Ratio
FuEn	Fuzzy Entropy
IES	Inverse Efficiency Score
IIR	Infinite Impulse Response

ISI	Inter-Stimulus Interval
MAD	Median Absolute Deviation
MFEI	Multiscale Fuzzy Entropy Index
MSEI	Multiscale Sample Entropy Index
PSD	Power Spectral Density
RT	Reaction Time
SaEn	Sample Entropy
tDCS	Transcranial Direct Current Stimulation
tES	Transcranial Electrical Stimulation
TNR	True Negative Rate
TPR	True Positive Rate

## References

[1] Sarter M., Givens B., Bruno J.P. (2001). The cognitive neuroscience of sustained attention: Where top-down meets bottom-up. Brain Res. Rev..

[2] Esterman M., Rothlein D. (2019). Models of sustained attention. Curr. Opin. Psychol..

[3] Walker H.E., Trick L.M. (2018). Mind-wandering while driving: The impact of fatigue, task length, and sustained attention abilities. Transp. Res. Part F Traffic Psychol. Behav..

[4] Vujic A. (2017). Switching on or switching off? Everyday computer use as a predictor of sustained attention and cognitive reflection. Comput. Hum. Behav..

[5] Rosengrant D., Hearrington D., O’Brien J. (2021). Investigating student sustained attention in a guided inquiry lecture course using an eye tracker. Educ. Psychol. Rev..

[6] Fortenbaugh F.C., DeGutis J., Esterman M. (2017). Recent theoretical, neural, and clinical advances in sustained attention research. Ann. N. Y. Acad. Sci..

[7] Brosnan M.B., Arvaneh M., Harty S., Maguire T., O’Connell R., Robertson I.H., Dockree P.M. (2018). Prefrontal modulation of visual processing and sustained attention in aging, a tDCS–EEG coregistration approach. J. Cogn. Neurosci..

[8] Qiao J., Li X., Wang Y., Wang Y., Li G., Lu P., Wang S. (2022). The Infraslow Frequency Oscillatory Transcranial Direct Current Stimulation Over the Left Dorsolateral Prefrontal Cortex Enhances Sustained Attention. Front. Aging Neurosci..

[9] Chiarion G., Sparacino L., Antonacci Y., Faes L., Mesin L. (2023). Connectivity Analysis in EEG Data: A Tutorial Review of the State of the Art and Emerging Trends. Bioengineering.

[10] Chiarion G., Mesin L. (2021). Functional Connectivity of EEG in Encephalitis during Slow Biphasic Complexes. Electronics.

[11] Clayton M.S., Yeung N., Kadosh R.C. (2015). The roles of cortical oscillations in sustained attention. Trends Cogn. Sci..

[12] Esterman M., Noonan S.K., Rosenberg M., DeGutis J. (2013). In the zone or zoning out? Tracking behavioral and neural fluctuations during sustained attention. Cereb. Cortex.

[13] Wascher E., Rasch B., Sänger J., Hoffmann S., Schneider D., Rinkenauer G., Heuer H., Gutberlet I. (2014). Frontal theta activity reflects distinct aspects of mental fatigue. Biol. Psychol..

[14] Khachouf O.T., Chen G., Duzzi D., Porro C.A., Pagnoni G. (2017). Voluntary modulation of mental effort investment: An fMRI study. Sci. Rep..

[15] Hilviu D., Vincenzi S., Chiarion G., Mattutino C., Roatta S., Calvo A., Bosco F.M., Gena C. Endogenous Cognitive Tasks for Brain-Computer Interface: A Mini-Review and a New Proposal. Proceedings of the International Conference on Computer-Human Interaction Research and Applications.

[16] Martins A.R., Fregni F., Simis M., Almeida J. (2017). Neuromodulation as a cognitive enhancement strategy in healthy older adults: Promises and pitfalls. Aging Neuropsychol. Cogn..

[17] Hyde J., Carr H., Kelley N., Seneviratne R., Reed C., Parlatini V., Garner M., Solmi M., Rosson S., Cortese S. (2022). Efficacy of neurostimulation across mental disorders: Systematic review and meta-analysis of 208 randomized controlled trials. Mol. Psychiatry.

[18] Coffman B.A., Clark V.P., Parasuraman R. (2014). Battery powered thought: Enhancement of attention, learning, and memory in healthy adults using transcranial direct current stimulation. Neuroimage.

[19] Filmer H.L., Dux P.E., Mattingley J.B. (2014). Applications of transcranial direct current stimulation for understanding brain function. Trends Neurosci..

[20] Lefaucheur J.P., Antal A., Ayache S.S., Benninger D.H., Brunelin J., Cogiamanian F., Cotelli M., De Ridder D., Ferrucci R., Langguth B. (2017). Evidence-based guidelines on the therapeutic use of transcranial direct current stimulation (tDCS). Clin. Neurophysiol..

[21] Bergmann T.O., Hartwigsen G. (2021). Inferring causality from noninvasive brain stimulation in cognitive neuroscience. J. Cogn. Neurosci..

[22] Nitsche M.A., Cohen L.G., Wassermann E.M., Priori A., Lang N., Antal A., Paulus W., Hummel F., Boggio P.S., Fregni F. (2008). Transcranial direct current stimulation: State of the art 2008. Brain Stimul..

[23] Caulfield K.A., Indahlastari A., Nissim N.R., Lopez J.W., Fleischmann H.H., Woods A.J., George M.S. (2022). Electric field strength from prefrontal transcranial direct current stimulation determines degree of working memory response: A potential application of reverse-calculation modeling?. Neuromodul. Technol. Neural Interface.

[24] Khadka N., Borges H., Paneri B., Kaufman T., Nassis E., Zannou A.L., Shin Y., Choi H., Kim S., Lee K. (2020). Adaptive current tDCS up to 4 mA. Brain Stimul..

[25] Caulfield K.A., George M.S. (2022). Optimized APPS-tDCS electrode position, size, and distance doubles the on-target stimulation magnitude in 3000 electric field models. Sci. Rep..

[26] San-Juan D., Morales-Quezada L., Garduño A.J.O., Alonso-Vanegas M., González-Aragón M.F., López D.A.E., Gregorio R.V., Anschel D.J., Fregni F. (2015). Transcranial direct current stimulation in epilepsy. Brain Stimul..

[27] Bornheim S., Thibaut A., Beaudart C., Maquet P., Croisier J.L., Kaux J.F. (2022). Evaluating the effects of tDCS in stroke patients using functional outcomes: A systematic review. Disabil. Rehabil..

[28] Pacheco-Barrios K., Cardenas-Rojas A., Thibaut A., Costa B., Ferreira I., Caumo W., Fregni F. (2020). Methods and strategies of tDCS for the treatment of pain: Current status and future directions. Expert Rev. Med. Devices.

[29] Woodham R., Rimmer R.M., Mutz J., Fu C.H. (2021). Is tDCS a potential first line treatment for major depression?. Int. Rev. Psychiatry.

[30] Nitsche M.A., Paulus W. (2000). Excitability changes induced in the human motor cortex by weak transcranial direct current stimulation. J. Physiol..

[31] McIntire L.K., McKinley R.A., Goodyear C., Nelson J. (2014). A comparison of the effects of transcranial direct current stimulation and caffeine on vigilance and cognitive performance during extended wakefulness. Brain Stimul..

[32] Thair H., Holloway A.L., Newport R., Smith A.D. (2017). Transcranial direct current stimulation (tDCS): A beginner’s guide for design and implementation. Front. Neurosci..

[33] Medina J., Cason S. (2017). No evidential value in samples of transcranial direct current stimulation (tDCS) studies of cognition and working memory in healthy populations. Cortex.

[34] Dedoncker J., Brunoni A.R., Baeken C., Vanderhasselt M.A. (2016). A systematic review and meta-analysis of the effects of transcranial direct current stimulation (tDCS) over the dorsolateral prefrontal cortex in healthy and neuropsychiatric samples: Influence of stimulation parameters. Brain Stimul..

[35] Bergmann T.O. (2018). Brain state-dependent brain stimulation. Front. Psychol..

[36] Fedotchev A., Parin S., Polevaya S., Zemlianaia A. (2021). Human body rhythms in the development of non-invasive methods of closed-loop adaptive neurostimulation. J. Pers. Med..

[37] Sudbrack-Oliveira P., Razza L.B., Brunoni A.R. (2021). Non-invasive cortical stimulation: Transcranial direct current stimulation (tDCS). Int. Rev. Neurobiol..

[38] Leite J., Morales-Quezada L., Carvalho S., Thibaut A., Doruk D., Chen C.F., Schachter S.C., Rotenberg A., Fregni F. (2017). Surface EEG-transcranial direct current stimulation (tDCS) closed-loop system. Int. J. Neural Syst..

[39] Sun M., yan Li H., Guo D. (2019). An adaptive transcranial direct current stimulation (tDCS). Proceedings of the 2019 6th International Conference on Systems and Informatics (ICSAI).

[40] Lustenberger C., Boyle M.R., Alagapan S., Mellin J.M., Vaughn B.V., Fröhlich F. (2016). Feedback-controlled transcranial alternating current stimulation reveals a functional role of sleep spindles in motor memory consolidation. Curr. Biol..

[41] Robinson C.S., Bryant N.B., Maxwell J.W., Jones A.P., Robert B., Lamphere M., Combs A., Al Azzawi H.M., Gibson B.C., Sanguinetti J.L. (2018). The benefits of closed-loop transcranial alternating current stimulation on subjective sleep quality. Brain Sci..

[42] Francisco-Vicencio M.A., Góngora-Rivera F., Ortiz-Jiménez X., Martinez-Peon D. (2022). Sustained attention variation monitoring through EEG effective connectivity. Biomed. Signal Process. Control.

[43] Wong H., Zaman R. (2019). Neurostimulation in Treating ADHD. Psychiatr Danub..

[44] Khaleghi A., Zarafshan H., Vand S., Mohammadi M. (2020). Effects of Non-invasive Neurostimulation on Autism Spectrum Disorder: A Systematic Review. Clin. Psychopharmacol. Neurosci..

[45] Antal A., Alekseichuk I., Bikson M., Brockmöller J., Brunoni A.R., Chen R., Cohen L., Dowthwaite G., Ellrich J., Flöel A. (2017). Low intensity transcranial electric stimulation: Safety, ethical, legal regulatory and application guidelines. Clin. Neurophysiol..

[46] Georgiou G., Essau C.A. (2011). Go/No-go task. Encyclopedia of Child Behavior and Development.

[47] Riccio C.A., Reynolds C.R., Lowe P., Moore J.J. (2002). The continuous performance test: A window on the neural substrates for attention?. Arch. Clin. Neuropsychol..

[48] Mueller S.T., Piper B.J. (2014). The psychology experiment building language (PEBL) and PEBL test battery. J. Neurosci. Methods.

[49] Townsend J., Ashby F., Castellan J., Restle F. (1978). Methods of Modeling Capacity in Simple Processing Systems.

[50] Kaushik P., Moye A., Vugt M.v., Roy P.P. (2022). Decoding the cognitive states of attention and distraction in a real-life setting using EEG. Sci. Rep..

[51] Welch P. (1967). The use of fast Fourier transform for the estimation of power spectra: A method based on time averaging over short, modified periodograms. IEEE Trans. Audio Electroacoust..

[52] Stam C.J. (2005). Nonlinear dynamical analysis of EEG and MEG: Review of an emerging field. Clin. Neurophysiol..

[53] Chen W., Zhuang J., Yu W., Wang Z. (2009). Measuring complexity using fuzzyen, apen, and sampen. Med. Eng. Phys..

[54] Costa M., Goldberger A.L., Peng C.K. (2005). Multiscale entropy analysis of biological signals. Phys. Rev. E.

[55] Pincus S.M. (1991). Approximate entropy as a measure of system complexity. Proc. Natl. Acad. Sci. USA.

[56] Richman J.S., Moorman J.R. (2000). Physiological time-series analysis using approximate entropy and sample entropy. Am. J. Physiol. Heart Circ. Physiol..

[57] Chen W., Wang Z., Xie H., Yu W. (2007). Characterization of surface EMG signal based on fuzzy entropy. IEEE Trans. Neural Syst. Rehabil. Eng..

[58] Azami H., Fernández A., Escudero J. (2017). Refined multiscale fuzzy entropy based on standard deviation for biomedical signal analysis. Med. Biol. Eng. Comput..

[59] Kosciessa J.Q., Kloosterman N.A., Garrett D.D. (2020). Standard multiscale entropy reflects neural dynamics at mismatched temporal scales: What’s signal irregularity got to do with it?. PLoS Comput. Biol..

[60] Mesin L. (2018). Estimation of Complexity of Sampled Biomedical Continuous Time Signals Using Approximate Entropy. Front. Physiol..

[61] Wan W., Cui X., Gao Z., Gu Z. (2021). Frontal EEG-based multi-level attention states recognition using dynamical complexity and extreme gradient boosting. Front. Hum. Neurosci..

[62] Lim S., Yeo M., Yoon G. (2019). Comparison between concentration and immersion based on EEG analysis. Sensors.

[63] Silva A.F., Zortea M., Carvalho S., Leite J., Torres I.L.d.S., Fregni F., Caumo W. (2017). Anodal transcranial direct current stimulation over the left dorsolateral prefrontal cortex modulates attention and pain in fibromyalgia: Randomized clinical trial. Sci. Rep..

[64] Magosso E., De Crescenzio F., Ricci G., Piastra S., Ursino M. (2019). EEG alpha power is modulated by attentional changes during cognitive tasks and virtual reality immersion. Comput. Intell. Neurosci..

[65] van Schouwenburg M.R., Zanto T.P., Gazzaley A. (2017). Spatial attention and the effects of frontoparietal alpha band stimulation. Front. Hum. Neurosci..

[66] Pamplona G.S.P., Heldner J., Langner R., Koush Y., Michels L., Ionta S., Salmon C.E.G., Scharnowski F. (2023). Preliminary findings on long-term effects of fMRI neurofeedback training on functional networks involved in sustained attention. Brain Behav..

